# An Observational Study of Glycopegylated Extended Half-Life Factor Prophylaxis in Hemophilia A and B in a Tertiary Care Center in India

**DOI:** 10.7759/cureus.79815

**Published:** 2025-02-28

**Authors:** Parul Bhatt, Harsh Patel

**Affiliations:** 1 General Medicine, GMERS Medical College and Hospital, Sola, Ahmedabad, IND; 2 Internal Medicine, GMERS Medical College and Hospital, Sola, Ahmedabad, IND

**Keywords:** extended half-life factors, hemophilia, india, individualization, real-world evidence

## Abstract

Prophylactic treatment for hemophilia in India remains significantly underutilized compared to developed nations, leading to reliance on episodic therapy with limited long-term benefits. This study evaluated the efficacy and safety of glycopegylated extended half-life (EHL) clotting factors in patients with hemophilia A and B. A retrospective observational design was employed, including 25 patients aged ≥18 years with moderate or severe hemophilia A or B who transitioned to EHL therapy. Hemophilia A patients (n=19) switched from standard half-life (SHL) factor VIII (FVIII) to weekly EHL FVIII prophylaxis, while hemophilia B patients (n=6) transitioned from on-demand SHL factor IX (FIX) to glycopegylated EHL FIX prophylaxis every 21 days. Outcomes were assessed through the annual bleeding rate (ABR), functional independence score in hemophilia (FISH score), and hemophilia joint health score (HJHS). A subset analysis for quality of life (QoL) was also performed on hemophilia A patients using the Haem-A-QoL questionnaire in patients with hemophilia A. Glycopegylated EHL FVIII therapy significantly reduced ABR and improved joint health and functional scores over 24 months (p<0.001). Hemophilia B patients demonstrated a significant reduction in ABR and joint bleeding rates at eight months (p<0.05). The study found that prophylaxis with individualized doses of glycopegylated EHL factors is not only effective in reducing bleeding episodes and improving joint health but also safe with improved treatment adherence, offering a viable prophylactic option in resource-constrained settings.

## Introduction

Hemophilia A and B are X-linked monogenic disorders caused by F8/F9 gene variants characterized by low levels of clotting factor VIII (FVIII)/factor IX (FIX), respectively [1]. According to the World Federation of Hemophilia (WFH) reported on the Annual Global Survey (AGS) 2023, India has the largest affected population (n=25,820) of hemophilia (hemophilia A; n=22,133 and hemophilia B; n=3,687) [2]. Globally, hemophilia treatment includes clotting factor replacement and non-factor therapies [3]. Current management strategies for hemophilia focus on two main pillars: on-demand (episodic therapy) and prophylaxis infusion of antihemophilic agents [2].

In India, prophylactic treatment for hemophilia A and B is low at 4% in patients more than 18 years of age, significantly less than the over 80% observed in developed countries, leading most patients to rely on episodic therapy [4]. Episodic therapy alleviates pain and bleeding but is insufficient to improve the overall bleeding profile or prevent long-term complications of hemophilia [5]. The World Federation of Hemophilia strongly recommends prophylaxis therapy in resource-constrained countries over episodic therapy. However, achieving factor trough levels higher than 1 IU/dL (1%) is challenging due to frequent infusion schedules with standard half-life (SHL) factors, which can lead to reduced patient adherence [6]. Additionally, while the ABR for patients receiving SHL therapy is often comparable to that of EHL therapy, the requirement of a higher infusion frequency associated with SHL imposes a greater economic burden on patients [7]. A Canadian study observed that the non-switchers (patients on SHL therapy) saw no overall change in the quality of life (QoL) and that 44% of them expressed a preference for EHL, in retrospect [8].

Extended half-life (EHL) therapies were developed to address these issues, improving adherence, promoting better health-related QoL, and reducing economic burden and bleeding-related hospitalization [9].

EHL factors serve as prophylactic therapy for individuals with hemophilia A and B [6]. EHL FVIII offers a half-life extension of 1.4-1.6 times, while EHL FIX shows an increase, extending three to five times longer than SHL counterparts [10,11]. This reduces the infusion frequency, typically once weekly for hemophilia B and twice weekly for hemophilia A, while providing more effective prophylaxis. This results in improved bleed prevention and adherence to similar dosing schedules. These improvements enhance the QoL for prophylaxis patients, particularly those with challenging venous access or frequent bleeding episodes [6].

The high cost of full-dose EHL therapies for hemophilia A and B, coupled with limited factor supplies and constrained healthcare budgets in resource-limited countries, necessitates the judicious use of these therapies to maximize patient benefits. As a result, even reduced doses of EHL are increasingly adopted, offering the potential for similar therapeutic benefits while alleviating the financial burden on patients [12]. Although existing literature primarily focuses on the standard dosing regimens, evidence suggests that reduced dosages of EHL can yield effective outcomes in managing hemophilia [13]. A study observed that patients receiving regular low-dose factor prophylaxis had lower annual bleeding rates (ABR) as compared to patients receiving episodic factor doses [14]. This approach can optimize treatment adherence and improve the overall QoL for patients, making it an attractive option for those limited by high medication costs.

Efficacy and safety outcomes of EHL FXIII or FIX prophylaxis for hemophilia A and B, respectively, have been studied in many other countries [10,11]. There are limited data from real-world practice for glycopegylated EHL prophylaxis regarding dose individualization in the Indian population. This study aims to evaluate the clinical outcomes and management strategies of hemophilia A and B patients receiving individualized glycopegylated EHL factor prophylaxis of FVIII/FIX therapy at a tertiary care center, addressing critical questions regarding effectiveness, safety, and patient QoL.

## Materials and methods

Study design

An observational, retrospective study was carried out in hemophilia A (n=19) patients who were switched from SHL FVIII (20 IU/kg thrice a week) prophylaxis to glycopegylated EHL (turoctocog alfa pegol) FVIII (50 IU/kg once a week) prophylaxis for 12 months. Hemophilia B patients (n=6) who were switched from FIX (20 IU/kg thrice a week) on-demand therapy to glycopegylated EHL (nonacog beta pegol) FIX (40 IU/kg once in 21 days) prophylaxis for eight months. The study was conducted at the Hemophilia Day Care Centre at GMERS Medical College and Hospital, Sola, Gujrat, India, from 2022 to 2024. Patient data were retrieved from the medical records (MR). The study was conducted following GCP guidelines and the Declaration of Helsinki and was approved by the Ethics Committee (approval no. GMERSMCS/IEC/77/2023).

Participant selection

This study included patients aged ≥18 years of age with moderate or severe hemophilia A or B who were receiving SHL FVIII prophylaxis or SHL FIX on-demand therapy and had an ABR of more than 10. Patients with a history of FVIII/FIX inhibitors were excluded. Patients with incomplete MR were excluded from the study.

Outcome measures

The effectiveness of glycopegylated EHL FVIII therapy was assessed by evaluating the overall joint bleed rate, spontaneous and traumatic bleed rates, and reduction in the ABR at six, 12, and 24 months from the baseline visit. Similarly, the effectiveness of glycopegylated EHL FIX therapy was assessed using the overall joint bleed rate, spontaneous and traumatic bleed rates, and the ABR eight months after the baseline visit. Joint health was evaluated using the hemophilia joint health score (HJHS). The functional independence was measured with the functional independence score in hemophilia (FISH), and an exercise/physiotherapy routine was also measured pre-treatment and post-treatment.

The resolution of bleeding episodes was recorded, with emphasis on the number of injections required for bleed cessation. A subset analysis (n=10) on QoL was done using the Haem-A-Qol questionnaire to see the difference between before and one year after EHL FVIII prophylaxis. Additional parameters, such as the assessment of target joint involvement and the occurrence of extra-articular bleeding episodes, were noted.

Statistical analysis

Descriptive statistics were employed to summarize the data, using measures such as mean, standard deviation (SD), median, and range. The Wilcoxon signed-rank test was applied to evaluate paired data, enabling comparison of changes within groups over time. A p-value less than 0.05 was considered statistically significant.

## Results

Demographics and baseline characteristics

In this observational retrospective study, a total of 16 patients were analyzed who were diagnosed with hemophilia A (n=19) and hemophilia B (n=6). The average age of patients was 33.05 (±10.61) in the hemophilia A group and 29.5 years (±10.3) in the hemophilia B group, while the mean weight was 71.21 (±13.09) and 64.2 kg (±4.76), respectively. Severe hemophilia was more common, observed in 94.6% of hemophilia A cases and 67% of hemophilia B cases. The median treatment duration for both groups was 12 months, with a median prophylactic dose of glycopegylated EHL FVIII at 50 IU/kg/week in hemophilia A and glycopegylated EHL FIX at 40 IU/kg every 21 days in hemophilia B. Common bleeding sites in hemophilia A included the right knee (21.81%), right ankle (10.90%), and right elbow (10.90%), whereas hemophilia B patients experienced frequent bleeding in both knees (25%) right knee (16.66%), left knee (16.66%), and left ankle (16.66%) (Tables 1-2).

**Table 1:** Demographic and baseline characteristics. n=number of patients, SD=standard deviation, IQR=interquartile range, EHL FVIII/IX=extended half-life factor VIII/IX, IU/kg=international unit per kilogram, and %=percentage of patients

Baseline Characteristics	Hemophilia A (N=19)	Hemophilia B (N=6)
Age; mean (SD)	33.05 (10.61)	29.5 (10.3)
Weight; mean (SD)	71.21 (13.09)	64.2 (4.76)
Severity of hemophilia: n (%)		
Severe	18 (94.6%)	4 (67%)
Moderate	1 (5.4%)	2 (33%)
Duration of treatment; median (IQR)	12.0 (6.0)	12.0 (5.0)
Dose of EHL FVIII/IX IU/kg; median (IQR)	50 (0.00)	40 (0.00)

**Table 2:** Percentage of bleeding site and target joints at baseline and after prophylaxis. n=number of patients, EHL=extended half-life factor, %=percentage of patients

Site	Bleeding site at baseline; n (%)		Target joints at baseline, n (%)		Target joints involved after EHL prophylaxis, n (%)	
	Haemophilia A (55)	Haemophilia B (12)	Haemophilia A (32)	Haemophilia B (10)	Haemophilia A (21)	Haemophilia B (6)
Right Ankle	6 (10.90%)	1 (8.33%)	5 (15.63%)	0 (0.00%)	4 (19.04%)	
Right Knee	12 (21.81%)	2 (16.66%)	8 (25.00%)	2 (20.00%)	8 (38.09%)	2 (33.33%)
Right Elbow	6 (10.90%)	0 (0.00%)	3 (9.37%)	0 (0.00%)	2 (9.52%)	1 (16.66%)
Right Shoulder	4 (7.26%)	0 (0.00%)	1 (3.12%)	0 (0.00%)	0 (0.00%)	0 (0.00%)
Left Elbow	5 (9.09%)	1 (8.33%)	4 (12.50%)	0 (0.00%)	0 (0.00%)	2 (33.33%)
Left Shoulder	1 (1.81%)	0 (0.00%)	0 (0.00%)	1 (10.00%)	0 (0.00%)	0 (0.00%)
Left Knee	5 (9.09%)	2 (16.66%)	3 (9.37%)	2 (20.00%)	3 (14.28%)	1 (16.66%)
Left Ankle	3 (5.45%)	2 (16.66%)	1 (3.12%)	0 (0.00%)	0 (0.00%)	0 (0.00%)
Left Hip	0 (0.00%)	1 (8.33%)	0 (0.00%)	0 (0.00%)	0 (0.00%)	0 (0.00%)
Both Elbows	2 (3.63%)	0 (0.00%)	1 (3.12%)	0 (0.00%)	1 (4.76%)	0 (0.00%)
Both Knees	5 (9.09%)	3 (25.00%)	5 (15.63%)	2 (20.00%)	3 (14.28%)	0 (0.00%)
Both Ankles	3 (5.45%)	0 (0.00%)	1 (3.12%)	0 (0.00%)	0 (0.00%)	0 (0.00%)
Right Wrist	1 (1.81%)	0 (0.00%)	0 (0.00%)	0 (0.00%)	0 (0.00%)	0 (0.00%)
Intracranial Bleed	2 (3.63%)	0 (0.00%)	0 (0.00%)	0 (0.00%)	0 (0.00%)	0 (0.00%)
No Target joint	0 (0.00%)	0 (0.00%)	0 (0.00%)	2 (20.00%)	0 (0.00%)	0 (0.00%)

Effectiveness outcomes

Hemophilia A

**Figure 1:**
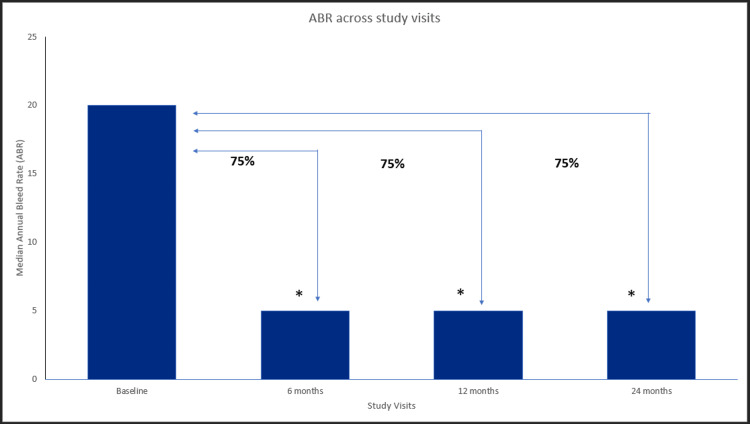
ABR across study visits in hemophilia A patients. Data were analyzed using the Wilcoxon signed-rank test. * indicates a statistically significant p-value (<0.05). ABR=annual bleeding rate

In patients with hemophilia A (n=19), treatment with EHL FVIII resulted in a 75.0% reduction in ABR score (median) from baseline (20) to 6 (5) months (p=3.81×10−6), 75.0% (5) at 12 months (p=3.81×10−6), and 75.0% (5) at 24 months (p=3.81×10−6) compared to the baseline (Figure 1). The overall joint bleeding rates (median) significantly decreased at 6 (3), 12 (5), and 24 (4) months from the baseline (20) (p<0.001). There was a significant reduction in the spontaneous bleeding rates (median) from baseline (2) to 6 (0), 12 (0), and 24 (0) months (p<0.001). There was a 100% reduction in traumatic bleeding rates (median) from baseline (1) to 6 (0), 12 (0), and 24 (0) months (p<0.001). 

After EHL FVIII prophylaxis in hemophilia A patients, the bleed resolution was achieved after one or two injections in 94.7% of patients (Table 3).

**Table 3:** Bleeding outcomes and joint involvement after EHL FVII/IX prophylaxis in hemophilia A and B patients. Y=yes, N=no, n=number of patients, %=percentage of patients, EHL=extended half-life

Parameters	Hemophilia A, n (%)	Hemophilia B, n (%)
Bleed Resolution; after 1 or 2 injections (Y/N)		
Y	18 (94.7%)	6 (100%)
N	1 (5.3%)	0 (0%)
Remaining bleed-free after EHL prophylaxis (Y/N)		
Y	1 (5.3%)	2 (33.33%)
N	18 (94.7%)	4 (66.66%)
Episodes of extra-articular bleeds after treatment started		
Y	1 (5.26%)	0 (0%)
N	18 (94.73%)	6 (100%)

**Figure 2:**
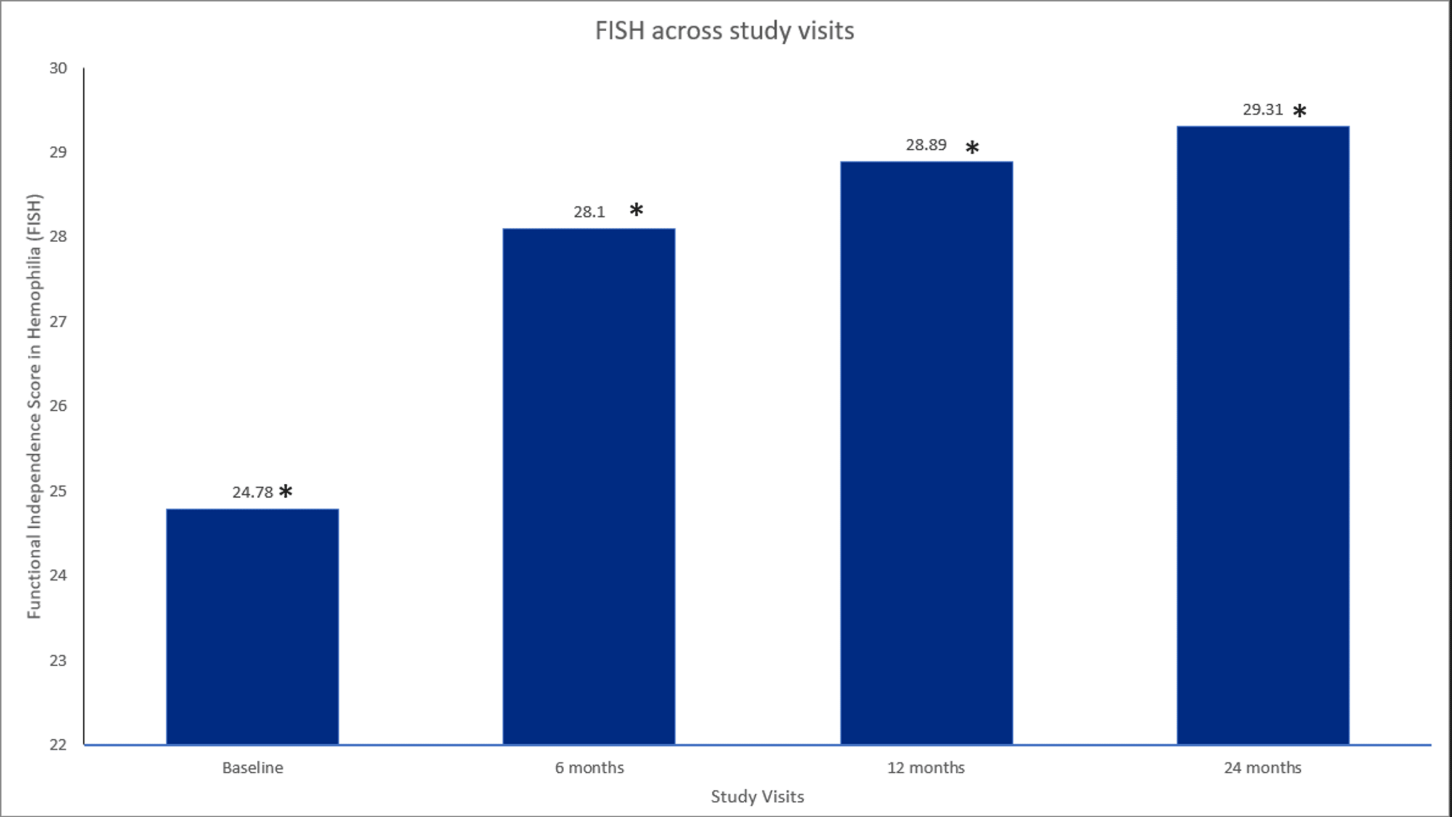
FISH across study visits in hemophilia A patients. Data were analyzed using the Wilcoxon signed-rank test. * indicates a statistically significant p-value (<0.05). FISH=functional independence score in hemophilia

**Figure 3:**
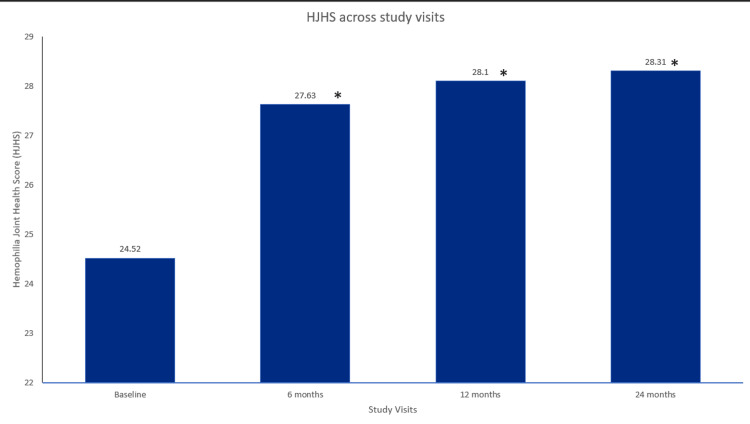
HJHS across study visits in hemophilia A patients. Data were analyzed using Wilcoxon signed-rank test. * indicates a statistically significant p-value (<0.05). HJHS=hemophilia joint health score

**Figure 4:**
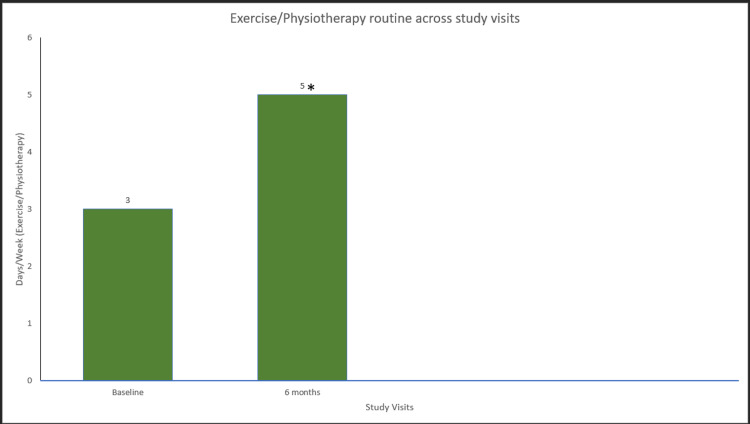
Exercise/physiotherapy routine days in hemophilia A patients. Data were analyzed using the Wilcoxon signed-rank test. * indicates a statistically significant p-value (<0.05).

Treatment with glycopegylated EHL FVIII in participants with hemophilia A also resulted in a statistically significant improvement in the mean FISH scores, showing a progressive improvement from baseline to 6 (p=0.00018), 12 (p=0.00019), and 24 (p=3.81×106) months, respectively (Figure 2). In addition, the mean HJHS score was significantly improved from baseline to six months (p=3.81×106), 12 months (p=0.00019), and 24 months (p=3.81×106) (Figure 3). Furthermore, hemophilia A participants treated with glycopegylated EHL FVIII showed a significant increase in the number of exercise/physiotherapy days per week from baseline to six, 12, and 24 months (p<0.001) (Figure 4).

**Figure 5:**
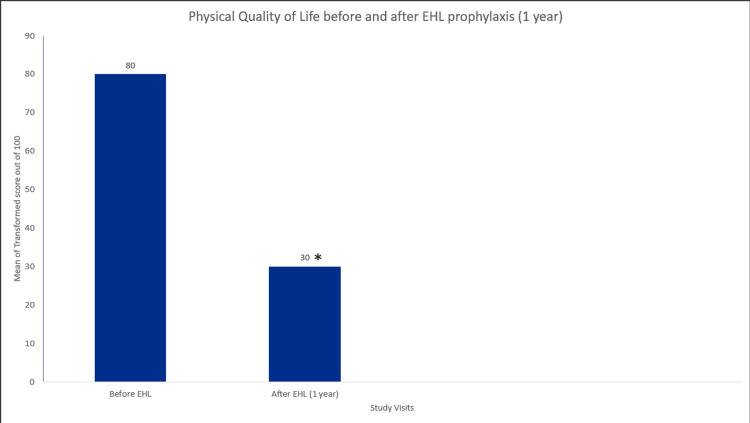
Quality of physical health score in hemophilia A patients. Data were analyzed using the Wilcoxon signed-rank test. * indicates a statistically significant p-value (<0.05).

**Figure 6:**
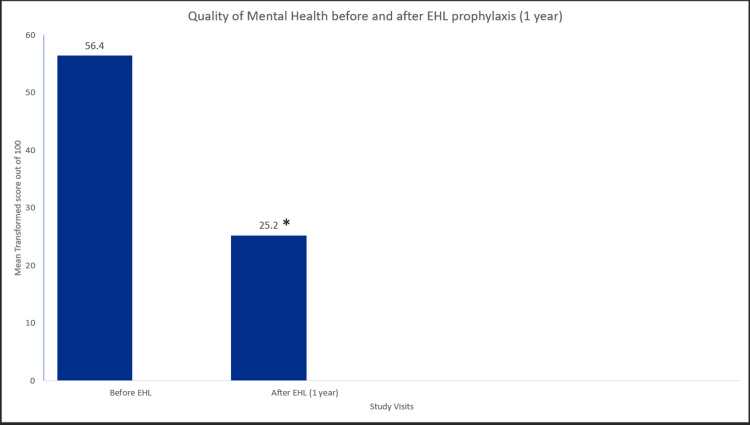
Quality of mental health score in hemophilia A patients. Data were analyzed using the Wilcoxon signed-rank test. * indicates a statistically significant p-value (<0.05).

A significant (p<0.005) difference was observed in both, the quality of physical health and quality of mental health (feelings related to hemophilia) after glycopegylated EHL FVIII prophylaxis of one year (Figures 5-6).

Hemophilia B

**Figure 7:**
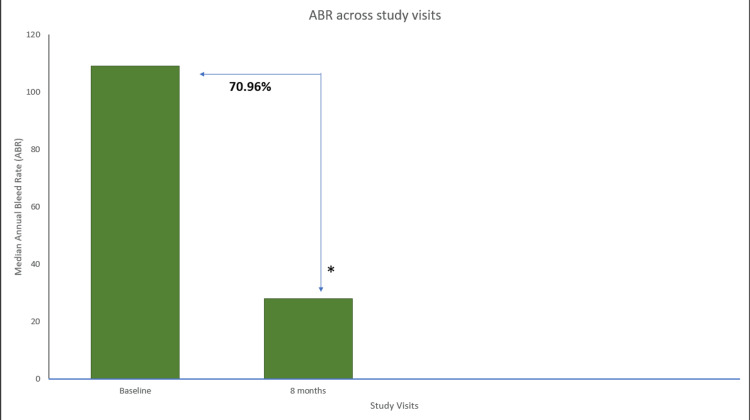
ABR across study visits in hemophilia B patients. Data were analyzed using Wilcoxon signed-rank test. * Indicates a statistically significant p-value (<0.05). ABR=annual bleeding rate

In the patients with hemophilia B (n=6), EHL FIX prophylaxis resulted in a 70.96% reduction in the median ABR score at eight months compared to the period before prophylaxis. The mean reduction in the ABR score was also found to be significant (p=0.031) (Figure 7). The overall joint bleeding rate was reduced by 84.22% in month eight from the baseline. The mean overall joint bleeding rate (p=0.031) and median spontaneous bleeding rate reduced by 80.64% and showed significant reduction (p=0.031) following eight months of 40 IU/kg/21 days of EHL FIX prophylaxis. However, an insignificant mean change was observed in the traumatic bleeding rate between the two time points (p=0.317). The traumatic bleeding rate remained consistently low at baseline (0) and 8 (0) months, and a statistically significant difference was not found (p>0.05)

After initiating glycopegylated EHL FIX prophylaxis in patients with hemophilia B, the bleed resolution was achieved after one or two injections in all patients. There was a 100% target joint bleed resolution observed in patients. All patients remained bleed-free, and no extra-articular bleeding episodes were reported in any of the patients (Table 3).

**Figure 8:**
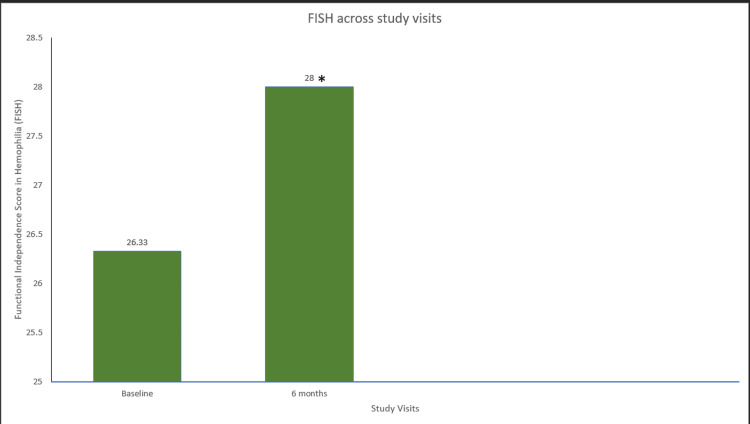
FISH across study visits in hemophilia B patients. Data were analyzed using the Wilcoxon signed-rank test. * indicates a statistically significant p-value (<0.05). FISH=functional independence score in hemophilia

**Figure 9:**
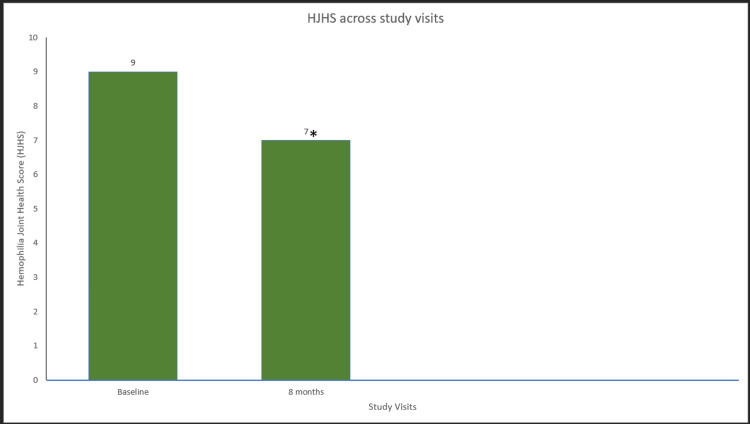
HJHS across study visits in hemophilia B patients. Data were analyzed using the Wilcoxon signed-rank test. * indicates a statistically significant p-value (<0.05). HJHS=hemophilia joint health score

**Figure 10:**
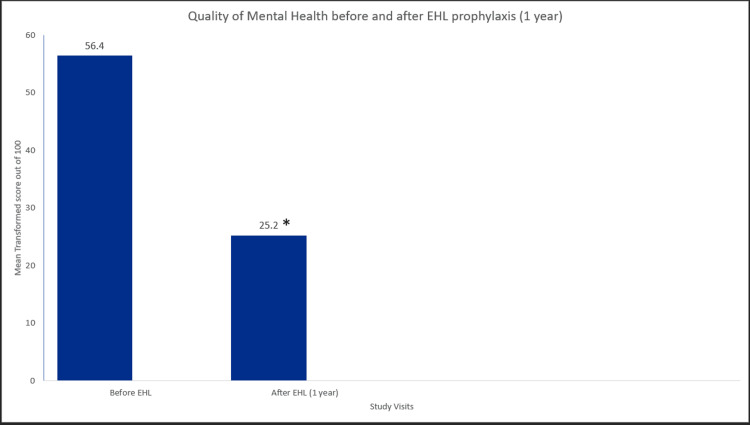
Exercise/physiotherapy routine days in hemophilia A patients. Data were analyzed using the Wilcoxon signed-rank test test. * indicates a statistically significant p-value (<0.05).

A significant improvement in the mean FISH scores was 28 at eight-month prophylaxis compared to baseline scores (26.33) (p=0.039) (Figure 8). In addition, the mean HJHS score was a statistically significant improvement after eight months of EHL FIX prophylaxis (p=0.041) (Figure 9). Furthermore, there was a statistically significant increase of 40% in the number of exercise/physiotherapy days between the two time points (p=0.042) (Figure 10).

Safety outcomes

There were no incidents of thrombophlebitis or any other infusion-related complications observed during the study. Furthermore, none of the hemophilia A (n=19) and hemophilia B (n=6) patients experienced any drug-related side effects or adverse drug reactions.

Treatment adherence was increased to 100%, and infusion rates were also reduced compared to SHL therapy (three to five times weekly) in patients with hemophilia A and hemophilia B. None of the patients developed inhibitors during the study.

## Discussion

The observational study demonstrated significant improvements in clinical outcomes for patients with hemophilia A and B following a switch to glycopegylated EHL FVIII/FIX prophylaxis. Key outcomes included reductions in the ABR, overall joint bleed rate, spontaneous and traumatic bleed rates, and marked improvements in joint health and functionality. These findings are consistent with existing literature that underscores the efficacy and safety of EHL therapies.

The results align closely with the Pathfinder phase III trial, which evaluated the efficacy of turoctocog alfa pegol (N8-GP, EHL FVIII) in preventing and treating bleeds in severe hemophilia A. In this trial, patients receiving N8-GP prophylaxis reported a median ABR for overall, joint, spontaneous, and traumatic of 0.00 (mean ABR: 3.70), with >50% experiencing no bleeds during the study. Moreover, the study demonstrated comparable safety and efficacy between weekly (Q7D) and every-four-day (Q4D) dosing regimens, with 58% of Q7D patients achieving zero bleeds and no inhibitor development [15]. Similarly, in our study, switching to glycopegylated EHL FVIII and FIX significantly reduced median ABR for overall, joint, spontaneous, and traumatic, further supporting the utility of this approach in managing hemophilia A.

Similarly, Sharma demonstrated the advantages of glycopegylated EHL factor therapy in 15 Indian patients. Patients previously on SHL therapies with an average ABR of 15.1 achieved a substantial reduction to a mean ABR of 1 after switching to EHL FVIII. This transition also eliminated spontaneous bleeding, reduced traumatic episodes, and improved patient QoL through increased physical activity and decreased absenteeism [16]. These studies, along with our findings, highlight the transformative impact of EHL FVIII on bleeding outcomes and patient well-being.

In hemophilia B, the Paradigm 2 phase III trial evaluated the efficacy of nonacog beta pegol (EHL FIX) at weekly doses of 10 and 40 IU/kg. Patients on the 40 IU/kg regimen reported a median ABR of 1.04, with an estimated mean ABR of 2.51 [17]. Similarly, the Paradigm 4 trial demonstrated safety and efficacy in patients aged 13-70 years, with median ABRs of 1.36 and 1.00 for 10 IU/kg and 40 IU/kg once-weekly dosing, respectively. These findings align with our study, where glycopegylated EHL FIX prophylaxis significantly reduced ABR in hemophilia B patients [18]. Notably, 100% of patients with hemophilia B in our cohort achieved bleed resolution within one or two injections, underscoring the consistency and effectiveness of this approach.

Our findings also demonstrated that 80% of hemophilia A patients achieved bleed resolution with just one or two prophylactic injections. Comparable results were observed in the Pathfinder phase III trial, where 83.6% of bleeds resolved with a single injection, and 95.5% resolved within two injections [15]. Similarly, nonacog beta pegol achieved a 94.6% overall success rate in treating bleeds, with 87.9% resolving after one injection, as reported in the Paradigm 2 trial [17]. This high success rate further supports the efficacy of EHL therapies in achieving rapid bleed control.

The transition to EHL therapies has also led to notable improvements in patient adherence and QoL. Existing literature supports these findings, as evidenced by the ASPIRE extension trial, which demonstrated improved joint health across all age groups in severe hemophilia A patients on prophylaxis [19]. For hemophilia B, prophylaxis with nonacog beta pegol 40 IU/kg significantly improved physical health and overall QoL, reinforcing the potential of EHL therapies to positively impact daily living.

Achieving an ABR close to zero or one in severe hemophilia A remains challenging despite advancements such as glycopegylated EHL factor therapies. High baseline bleeding rates, driven by severe clotting factor deficiencies and frequent spontaneous bleeds, complicate treatment outcomes. Additionally, the short duration of follow-up in clinical studies often limits the ability to capture meaningful long-term ABR reductions. While EHL therapies offer improved pharmacokinetics- baseline severity, trauma-related bleeds, and limited observation periods hinder the achievement of near-zero ABR levels. Inadequate or delayed prophylaxis during earlier life stages can also affect the probability of achieving near-zero ABR levels [20].

Recognizing the heterogeneity in patient phenotypes and bleeding patterns is essential for optimizing hemophilia management. Individualized treatment plans that consider factors such as lifestyle, joint health, and bleeding phenotype can lead to better outcomes and improved adherence. Shared decision-making (SDM), as discussed by Valentino et al., emphasizes tailoring therapies to align with patient needs and preferences [21]. Mancuso et al. and Coppola et al. similarly advocate for personalized prophylactic strategies, particularly in children, to reduce long-term complications and enhance QoL [22,23].

The use of extended half-life therapies, particularly at reduced dosages, has demonstrated cost-effectiveness compared to SHL therapies. A Spanish study has demonstrated that patients utilizing EHL products report fewer annual infusions and lower overall factor consumption without compromising treatment efficacy, ultimately leading to significant cost savings. Patients transitioning from SHL to EHL therapies have experienced an average savings of approximately €11,227 per year due to the reduced frequency of dosing [24]. The maintenance of low ABRs indicates that reduced EHL regimens can be just as effective as traditional dosing, providing patients with excellent outcomes while minimizing treatment costs [12,14]. Our study corroborates this finding, as we achieved significant reductions in ABR with a lower-than-standard dose of 50 IU/kg administered every four days (50 IU Q4D). Since there are limited data on the applicability of these findings within the Indian population, this study contributes unique, much-needed insights into the benefits and efficacy of reduced EHL dosages.

Despite these promising findings, the present study has certain limitations. The retrospective and observational design introduces the potential for selection bias, and the relatively small sample size limits the generalizability of the results. Additionally, the study's follow-up period may not fully capture the long-term effects of EHL therapies. Future randomized controlled trials with larger cohorts and extended follow-up periods are needed to validate and expand upon these findings.

In conclusion, the administration of glycopegylated EHL FVIII at 50 IU/kg weekly or EHL FIX at 40 IU/kg every 21 days has demonstrated substantial improvements in bleeding outcomes, joint health, and functional independence in patients with hemophilia A and B. The significant reduction in ABR, coupled with enhanced QoL and adherence, underscores the potential of EHL therapies as a transformative approach to prophylactic treatment. These findings advocate for broader implementation of EHL prophylactic treatments to optimize care and improve the QoL for individuals living with hemophilia. Future research should focus on larger-scale studies to confirm these promising results and explore long-term impacts on patient health and adherence.

## Conclusions

This study demonstrates that glycopegylated EHL FVIII and EHL FIX prophylaxis significantly improve clinical outcomes in patients with hemophilia A and B. The substantial reductions in ABRs, along with enhanced joint health and functional independence, highlight the efficacy and safety of these therapies in a resource-constrained setting. Furthermore, the high adherence rates achieved with individualized dosing schedules suggest that EHL therapies can effectively address the treatment gaps prevalent in India. Additionally, low-dose prophylaxis with FVIII (50 IU/kg) has proven to be highly effective in overall health improvements, enabling broader access to treatment for more patients despite the limited availability of EHL factors.
